# Socioeconomic vulnerability and the management of domestic animal hosts in urban environments: a one health issue

**DOI:** 10.1186/s12917-025-05062-7

**Published:** 2025-12-13

**Authors:** Ianei de Oliveira Carneiro, Thaís Auxiliadora dos Santos Mattos, Simone Martins Freitas, Nicole Hlavac, Mike Begon, Federico Costa, Hussein Khalil, Hernan Darío Argibay

**Affiliations:** 1 Departamento de Medicina Veterinária Preventiva e Produção Animal, Escola de Medicina Veterinária e Zootecnia, Universidade Federal da Bahia, Salvador, Brasil; 2 Instituto Gonçalo Moniz, Fundação Oswaldo Cruz, Ministério da Saúde, Salvador, Brasil; 3 Clínica Veterinária, Universidade Salvador (UNIFACS), Salvador, Bahia Brasil; 4 Departamento de Anatomia, Patologia e Clínicas Veterinárias, Escola de Medicina Veterinária e Zootecnia, Universidade Federal da Bahia, Salvador, Bahia Brasil; 5 School of Biological Sciences, University of Liverpool, Crown Street, Liverpool, UK; 6 Instituto de Saúde Coletiva, Universidade Federal da Bahia, Salvador, Brazil; 7 Department of Wildlife, Fish, and Environmental Studies, Swedish University of Agricultural Sciences, Uppsala, Sweden

**Keywords:** Urban animals, Public health, Veterinary epidemiology

## Abstract

**Background:**

Within socioeconomically vulnerable communities, dogs and cats are also exposed to deficient sanitation infrastructure, conditions of mistreatment, and malnutrition. This scenario promotes the maintenance of pathogen reservoirs, posing risks to domestic and wild animals, as well as to humans. Considering this context, our objective is to demonstrate how socio-environmental conditions influence the reduction in quality of life and the vulnerability status of these animals, making them more prone to infections and the transmission of zoonotic pathogens. In a cross-sectional study, we applied questionnaires to volunteer pet guardians in the neighborhoods of Marechal Rondon (MR) and Pau da Lima (PDL), Salvador, Bahia, Brazil, and collected blood and stool samples from dogs and cats for hematological and parasitological studies. A population of dogs and cats treated at a private veterinary service was used as a control for hematological analyses. Statistical analysis of the variables of interest was performed using univariate mixed generalized linear models, multimodel inference, and quantitative model classification based on Akaike's Information Criterion (AIC) and the weighting of the relative contribution of each variable to the average model.

**Results:**

We sampled 202 dogs and 128 cats in MR and 132 dogs and 42 cats in PDL. Among the dogs, 242 underwent blood counts, and 137 underwent stool parasitology tests. Among the cats, 96 underwent blood counts, and 30 underwent parasitology tests. We observed significant differences in the average values of HCT, PPT, and Eos between animals from the communities and the control group, both for dogs and cats. Different individual animal variables, household characteristics, and environmental factors were associated with changes in hematological and parasitological parameters, thus affecting the overall health of the dogs and cats.

**Conclusions:**

This study highlights the need for basic animal health measures, such as sterilization, improved nutrition, deworming, and controlling street access, to reduce the competence of these animals as hosts of infectious agents, considering the vulnerability of these communities. Therefore, it is necessary to expand public policies focused on the promotion and prevention of comprehensive health, extending these measures to animal health.

**Supplementary Information:**

The online version contains supplementary material available at 10.1186/s12917-025-05062-7.

## Background

Urbanization poses a significant challenge within the framework of One Health, primarily due to various interfaces that facilitate the transmission of zoonotic diseases [1]. Environmental, socio-cultural, and economic factors create a continuous bidirectional process of host-pathogen interaction [2]. Factors such as the presence of garbage, flooding, and inadequate sewage systems, combined with the presence of stray and synanthropic animals, are typically associated with socioeconomically and environmentally vulnerable populations, thereby increasing the risk of spillover events from domestic animals to humans [3]. For several neglected tropical diseases, domestic animals (in particular dogs) play a crucial role in the maintenance and transmission of the disease transmission, for example, Chagas disease, Leishmaniasis, Rabies and Echinococcosis, and low general health status and coinfection with multiple parasites may further increase their host competence [4].

In vulnerable urban communities from low- and middle-income countries, it is not uncommon to find animals deprived of a proper diet, lacking vaccinations and parasite control, and subjected to conditions of mistreatment. This situation raises concerns about both animal welfare and One Health [5]. Being more exposed to stressful and immunosuppressive factors such as malnutrition, violence, and deprivation, may lead these animals to a higher risk of infection and enhance their host competence [6]. Also, they may act as sentinels, indicating environmental contamination and the circulation of pathogens between human and non-human animal populations [7]. Given that they face the same environmental and social adversities as humans, dogs and cats, depending on their management, become a crucial link in the epidemiology of infectious diseases in such areas [8]. This reinforces the importance of including these animals in the development of prophylactic measures, public animal health and welfare policies, and zoonosis control.

Given this scenario, a multidisciplinary approach is essential to understand the complex human-animal-environment interactions underlying the health-disease profile of these communities. Applying the One Health concept allows us to recognize the influence of each element and the importance of viewing these animals as integral to care, rather than merely instrumentalizing them or placing responsibility on them [9]. There is a critical need to discuss the socio-environmental factors influencing the health of dogs and cats residing in economically disadvantaged communities, and the consequential impact of zoonotic disease transmission on health.

The association between animal health and poverty, through the analysis of basic parameters (parasitological and hematological), may contribute to an understanding of how this scenario promotes the maintenance of hosts and impacts both human and animal well-being [10, 11]. Hematological indicators, including hematocrit, leukocyte counts, and eosinophilia, serve as sensitive biomarkers of physiological status in animals. Variations in these parameters can reflect a wide range of health alterations, from metabolic and nutritional imbalances to immune responses triggered by pathogen exposure or environmental stressors [11]. Because they provide an integrative view of host condition, such indicators are particularly valuable for sanitary assessments, offering insights into both individual health and broader population-level dynamics. Reduced hematocrit levels and/or anemia may reflect chronic malnutrition, iron and cobalamin deficiencies, or ongoing parasitic and infectious diseases that can cause chronic blood loss. These conditions are particularly prevalent in socioeconomically vulnerable settings, where poor nutritional status and limited access to veterinary care and preventive medicine exacerbate health risks [12]. Eosinophilia is a well-established marker of helminthic infections and allergic processes, and when associated with chronic parasitism, stress, or malnutrition, it reflects impaired immune competence and increased susceptibility to secondary infections [13]. Coproparasitological evaluation offers critical evidence of environmental exposure and pathogen circulation, as the presence of intestinal parasites in feces reflects active community transmission. Species such as hookworms (*Ancylostoma* spp.) and *Toxocara* spp. impair animal health through anemia, eosinophilia, and malnutrition, while also posing major zoonotic risks. In urban areas with poor sanitation, inadequate waste management, and free-roaming dogs and cats defecating in public spaces, coproparasitology becomes a critical indicator of both animal vulnerability and public health threats. Combined with hematological findings, these assessments illustrate how socio-environmental adversity compromises animal health and increases reservoir competence for zoonotic pathogens [12].

In this study, we aim to assess socio-environmental factors and husbandry practices associated with health features of dogs and cats in two urban communities in Salvador, Bahia, northeastern Brazil. By analyzing basic hematological parameters and conducting coproparasitological examinations, we seek to assess how socio-environmental conditions correlates with the quality of life and vulnerability of these animals, making them more susceptible to infections and perpetuating a vicious cycle between health parameters and burden of infectious pathogens [12]. Thus, the increased transmission of zoonotic pathogens, may enhance the persistence of human infectious diseases within these communities.

## Methods

### Study area

**Fig. 1:**
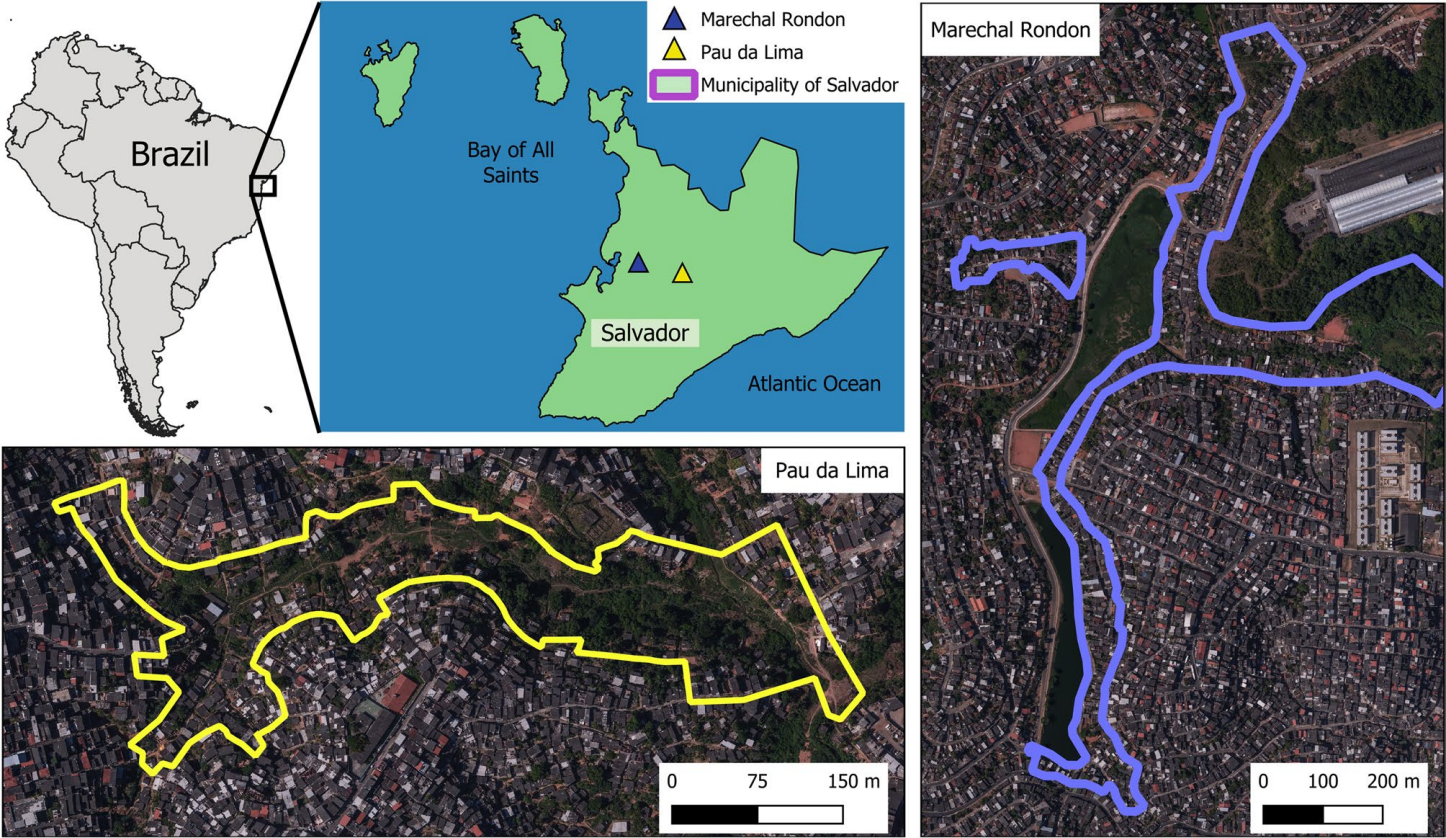
Distribution of the study area. The blue polygon outlines the study area within the Marechal Rondon neighborhood. The yellow polygon represents the Pau da Lima neighborhood, both located in Salvador, Bahia, Brazil

**Fig. 2:**
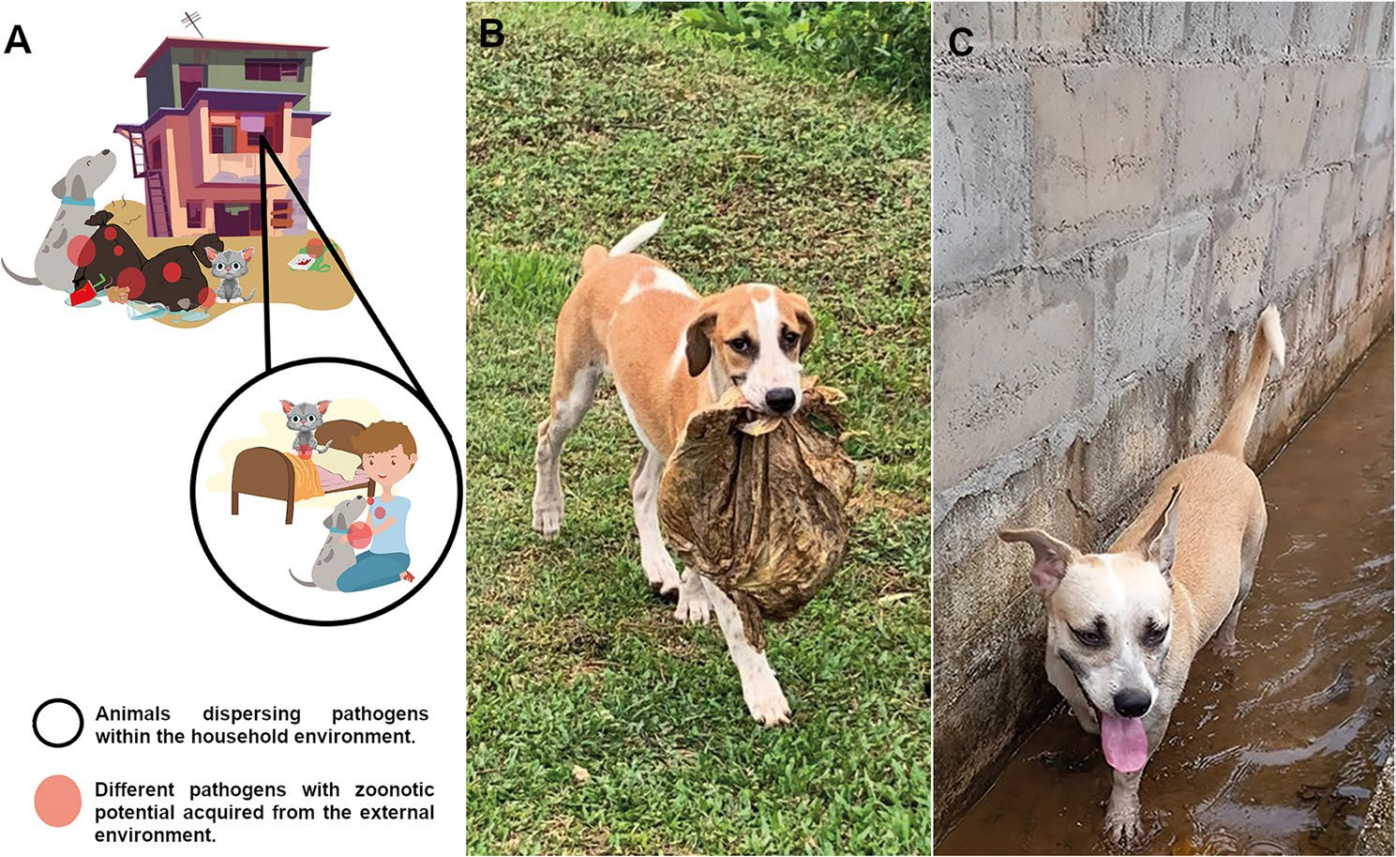
Flow of Pathogen Dispersal Between Humans and Animals in Vulnerable Urban Communities. **A** The red circles represent pathogens. The circles outlined with black lines indicate the interaction environment where these pathogens are dispersed. Semi-domesticated dogs and cats come into contact with garbage, sewage, and vectors, and then return home, which facilitates the transfer of pathogens to humans during interactions. Illustration created using CorelDRAW® v. 2022 with free images from Freepik. **B** Semi-domiciled dog from the community carrying a bag of garbage in its mouth. **C** Semi-domiciled dog from the community walking in a stream

We conducted a cross-sectional study in the neighbourhoods of Marechal Rondon (MR) and Pau da Lima (PDL) in Salvador, Bahia, northeastern Brazil (Fig. 1). Both areas exhibit significant socioeconomic vulnerability, categorized as subnormal settlements, with poor sanitary infrastructure, a history of flooding, and the presence of previously described zoonotic pathogens. These neighbourhoods also have high rates of violence, population density [14], and a large number of stray and semi-domiciled dogs and cats, as well as domiciled animals living under vulnerable conditions. This situation highlights the potential for pathogen transmission between humans and animals (Fig. 2).

### Recruiting and Obtaining Samples

The study areas within the neighbourhoods were selected as part of an eco-epidemiological project aimed to build and implement participatory interventions to improve environmental and human health [15]. The criteria to define the study areas was based on predefined vulnerability criteria: households located within 30 m of a sewer or dyke, with a history of flooding, the presence of garbage accumulation sites, and poor infrastructure. Within the delineated study areas of MR and PDL, 1125 and 334 premises, respectively, were identified and georeferenced. All premises in MR studied area were visited between November 2021 and May 2022, and all those in PDL between January and February 2023. During visits, once the presence of domestic animals in the house was confirmed, the guardians were personally invited to participate. Using ratio expansion from household censuses conducted in the parallel epidemiological survey, we estimated dog and cat populations. In MR, 472 of 1125 households were surveyed, yielding 219 dogs and 178 cats, corresponding to estimated totals of 523 dogs and 215 cats. In PDL, 130 of 334 households were surveyed, yielding 94 dogs and 52 cats, with estimated totals of 241 dogs and 133 cats. Refusal was defined when three consecutive visits were conducted without obtaining a response, when no animals were present at the residence, or when a lack of interest in participating in the project was expressed.

**Fig. 3:**
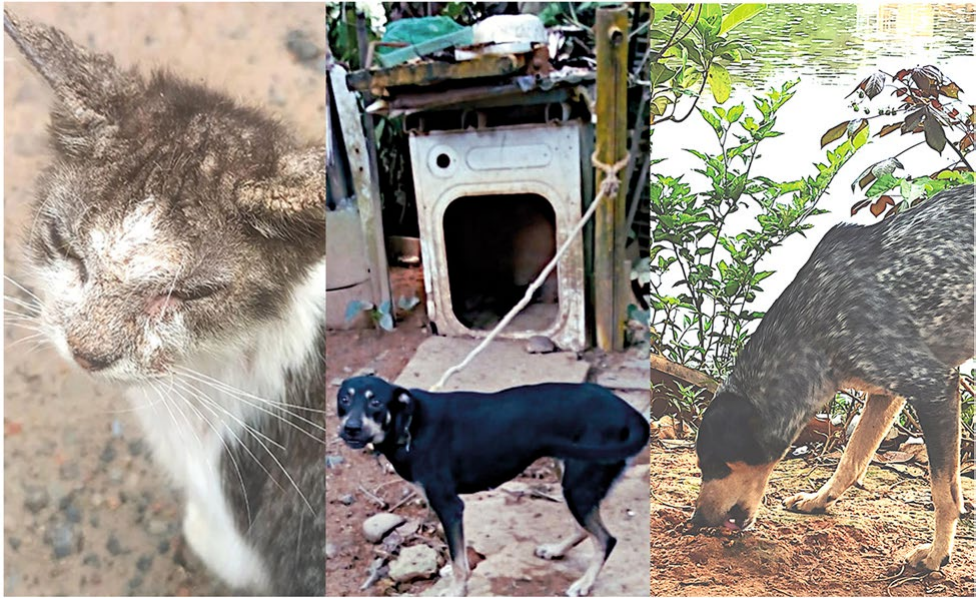
Clinical and management history of dogs and cats in participating communities. (left) Semi-domesticated cat affected by scabies dermatitis; (center) Dog kept on a leash in the peridomestic environment in precarious conditions; (right). Semi-domiciled dog eating leftover food straight off the floor

Project information was provided and the Free and Informed Consent Form (FICF) was signed. Oral interviews were then conducted using a pre-designed semi-structured questionnaire (see additional file 1). This questionnaire included inquiries about general management, food (access to the street, type of food), hygiene, and overall health conditions (deworming, sterilization, health history, vaccinations, presence of parasites) (Fig. 3). The collected data were then stored in REDCap^®^.

The research team prioritized gentle and respectful handling of the animals to ensure their well-being. This included using appropriate towels for physical restraint of cats and rewarding dogs after procedures. For safety and hygiene, dogs were fitted with disposable nylon muzzles. In cases where dogs were difficult to handle, collecting whole blood was not feasible. Reactive cats received a single intramuscular (IM) injection of ketamine (3 mg/kg) combined with dexmedetomidine (10 µg/kg). At the end of the procedure, atipamezole (250 µg/kg, IM) was administered [16, 17]. Prior to venipuncture in the cephalic or jugular vein, with 22 and 24 Gauge hypodermic needles, all animals underwent a general clinical examination to assess respiratory and heart rates, mucous membrane color, body condition, hydration status, and temperature. Approximately 2 mL of whole blood was collected into tubes manufactured with K₂EDTA anticoagulant (final concentration of 1.5 mg/mL of blood in vacuum collection tubes) and refrigerated for subsequent hematological analysis.

The hemogram included a complete blood count (CBC), performed using a veterinary automated hematology analyzer (URIT 5160 Vet, URIT Medical Electronic Co., Ltd., Guilin, China), along with hemoglobin determination. White blood cell (WBC) differentials and morphological assessments were conducted on Romanowsky-stained blood smears. In cases where platelet aggregates were observed, automated counts were disregarded and platelet numbers were determined manually from blood smears. All hematological analyses were performed on the same day as collection at the Veterinary Clinical Analysis Laboratory (LAC) of the Veterinary Hospital, School of Veterinary Medicine and Animal Science, Federal University of Bahia (EMEVZ-UFBA), using routine reference intervals reported in the literature for each species. In addition, we compared the haematological values of the sampled population with those of a reference group (REF), consisting of 133 apparently healthy dogs and 67 apparently healthy cats, admitted to a private veterinary hospital during the same period for elective non-infectious procedures or routine vaccination. This veterinary hospital, which belongs to a veterinary sciences private school, is located in the Imbuí neighborhood of Salvador, Bahia, Brazil, a residential area of middle to upper-middle socioeconomic status [18]. Animals were included in the control group only if they presented for elective, non-infectious procedures (primarily sterilization) or vaccination, and were considered clinically healthy based on the admission physical examination conducted by the local veterinarian. This examination included assessment of vital parameters, hydration, body condition, and absence of systemic or infectious disease. Blood collection was performed following a methodology similar to that used in the study, regarding the access route and sample handling. Complete blood counts were obtained using an automated hematology analyzer (BC 2800 Vet, Mindray Bio-Medical Electronics Co., Ltd., Shenzhen, China). Animals with ongoing infectious, metabolic, or chronic conditions were excluded. This group was selected as a reference because it comprises animals in general from outside the study communities, predominantly companion animals maintained under stable conditions of nutrition, hygiene, and preventive healthcare. As such, they provide a suitable baseline for comparison with the study population, which resided in the socio-environmentally vulnerable neighborhoods of Marechal Rondon and Pau da Lima, where suboptimal living conditions may directly influence animal health.

Faeces were collected either by team members upon finding fresh samples in the environment or by the pet guardians when none were available. A superficial portion of feces was collected and placed in containers with 5% formaldehyde, then sent for analysis using the flotation technique with a sugar-saturated solution (density of ~ 1.3 g/mL) [19], followed by optical microscopy (40X) (Nikon Eclipse Ei R, Tokyo, Japan).The Willis method was chosen due to its efficiency, simplicity, and ability to diagnose helminth eggs and larvae (geohelminths) as well as coccidian oocysts [20].

### Variables and statistical analysis

Variables obtained from the cross-sectional study were grouped into the following categories. (i) Individual Characteristics, including information on the species, sex, age, sterilization status, type of food, management practices, shelter conditions, vaccination history, and deworming status of the animals. (ii) Household Environment, with variables in this category encompassing environmental factors such as neighbourhood data, presence of peridomiciliary areas, paving conditions, garbage disposal methods, sewage infrastructure, altitude, and the percentage of ground cover within 20 and 50 m radius from the home. (iii) Guardian Sociodemographics, covering the living conditions of the participants, including factors such as food insecurity, number of residents per room, and the type of construction material used for the house.

To ensure meaningful correlations, we focused on key hematological variables that can indicate the physiological and clinical condition of the animals. These variables were chosen specifically to correlate with household environmental and sociodemographic factors. They include hematocrit (HCT), total protein (PPT), leukocyte count (WBC), neutrophil count (Neu), lymphocyte count (Lym), and eosinophil count (Eos). Regarding stool parasitology, the presence of hookworm was chosen as the variable for correlation. This parasite is the most prevalent genus among dogs in Brazil [21] and is a neglected geohelminthiasis with zoonotic potential, primarily transmitted through skin contact with contaminated soil [22, 23]. Its prevalence is closely associated with poverty, inadequate hygiene, limited access to water, and insufficient preventive measures and education [24].

For the statistical analysis, we initially conducted univariate generalized linear mixed models and selected variables with a significance level of *p* < 0.1. These chosen variables were subsequently included in multivariate generalized linear models. Although the inclusion of household as a random effect was tested, no significant relevance was observed.

To address collinearity, we assessed the variance inflation factor (VIF) for all variables in the comprehensive model, retaining only those with VIF < 4. Model selection was then performed using a multimodel inference approach [25]. This method allows for the quantitative ranking of models based on the Akaike information criterion (AIC), and it calculates the relative contribution of each variable in the averaged model. The procedure involved estimating the parameters for all possible combinations of independent variables and determining the relative importance (RI) of each variable. RI was calculated as the sum of Akaike weights across all models containing that variable, with values from 0 to 1, where 1 indicates maximum relative importance among the variables considered. To provide a descriptive overview of the relevance of each of each predictor variable on the health of the dogs and cats in the communities, we summed the weights of the variables and ranked them accordingly. The analyses were conducted using R version 3.2.3 (R Core Team, 2015) with the “MuMin” package.

In addition, to observe trends in the parameters assessed between the study population and a reference group, we compared the haematological results observed in the MR and PDL animals with a reference group. To assess the statistical difference in the parameters between both populations we performed Mann Whitney analysis.

## Results

In MR, 202 of the estimated 523 dogs (38%) and 128 of the estimated 215 cats (30%) were sampled, while in PDL, 132 of the estimated 241 dogs (54%) and 43 of the estimated 133 cats (32%) were sampled. Among the dogs, 242 underwent a blood count, and 137 underwent a stool parasitology test. Among the cats, 96 underwent a blood count, and 30 underwent a parasitology test.

In comparing the community animals with our reference, control population (Table 1), we observed that hematocrit levels were lower in community dogs (41.88 ± 8.56 vs. 44.69 ± 7.99) and cats (36.02 ± 5.83 vs. 39.52 ± 5.64), while total protein values (g/dL) were higher in community dogs (8.2 ± 1.3 vs. 6.57 ± 1.06) and cats (7.74 ± 0.78 vs. 6.39 ± 0.87). WBC counts (/µL) were significantly higher (*p* < 0.01) in community cats compared to control cats (15354.55 ± 7406.8 vs. 11535.82 ± 3928.44), and while higher WBC counts were also observed in community dogs, the difference was not statistically significant. For neutrophil counts (Neut) (/µL), a significant reduction (*p* < 0.04) was noted in community dogs compared to control-group dogs (6281.11 ± 3004.77 vs. 6584.71 ± 2373.41), with similar trends observed in cats, although statistical significance was not reached. The lymphocytes (Linf,/µL) of community cats were significantly increased (*p* < 0.01) compared to the control group (4936.86; ± 2489.28 vs. 3410.72; ± 2322.21), though the increase in this variable observed in community dogs was not statistically significant. And finally, the eosinophils (Eos,/µL) of both community dogs (1358.89; ± 1261.79 vs. 309.98; ± 338.21) and cats (638.45; ± 583.58 vs. 1194.62; ± 1034.27) showed significant differences when compared to the control groups, both with *p* < 0.01.

**Table 1:** Comparison of hematological values of dogs and cats sampled in marechal Rondon (MR) and Pau Da Lima (PDL) communities vs. Animals from the reference population. Significant differences are indicated in bold

	Variable	*N*° of sampled animals (MRC)	*N*° of sampled animals (REF)	Mean (sd) of animals from MR and PDL	SSA Mean (sd) of animals from Clivet	W	*p*
Canine	HCT (%)	242	133	41.88 (8.56)	44.69 (7.99)	28050.5	<0.01
	PPT (g/dL)	216	133	8.2 (1.3)	6.57 (1.06)	13093.5	<0.01
	WBC (/µL)	243	133	11115.84 (4624.54)	10160.65 (2534.78)	23,813	0.21
	Neut (/µL)	243	133	6281.11 (3004.77)	6584.71 (2373.41)	27126.5	0.04
	Linf (/µL)	243	133	3074.72 (2515.1)	2757.89 (1728.45)	24,385	0.5
	Eos (/µL)	242	133	1358.89 (1261.79)	309.98 (338.21)	13724.5	<0.01
Feline	HCT (%)	76	67	36.02 (5.83)	39.52 (5.64)	5710.5	<0.01
	PPT (g/dL)	65	67	7.74 (0.78)	7.45 (0.86)	4825.5	0.02
	WBC (/µL)	77	67	15354.55 (7406.8)	11535.82 (3928.44)	4112	<0.01
	Neut (/µL)	77	67	8546.51 (6041.71)	7148.3 (3259.64)	4703	0.54
	Linf (/µL)	77	67	4936.86 (2489.28)	3410.72 (2322.21)	3875.5	<0.01
	Eos (/µL)	77	67	1194.62 (1034.27)	638.45 (583.58)	3944.5	<0.01

*REF Reference population

In assessing the association between individual and household factors and hematological parameters (Table 2), variables contributing to a decrease in haematocrit (HCT) in dogs included senility [coef = −4.29 (95% CI: −7.65 to −0.94)], sheltering outside the home [coef = −6.19 (95% CI: −12.09 to −0.3)], consumption of homemade food [coef = −3.93 (95% CI: −6.5 to −1.36)] compared to commercial feed, and semi-domiciliary management [coef = −2.97 (95% CI: −5.11 to −0.84)]. Dogs living in Pau da Lima [coef = 4.65 (95% CI: 2.41 to 6.88)] and with a higher percentage of impermeable ground cover within a 50 m radius [coef = 0.13 (95% CI: 0.04 to 0.21)] were more likely to have increased HCT. In cats, significant increases in HCT were observed only with sterilization [coef = 4.7 (95% CI: 2.11 to 7.29)] and living away from open sewers [coef = 0.02 (95% CI: 0 to 0.03)].

**Table 2:** Association between individual and household factors and hematological parameters of canines and felines in communities

	Canines		Felines	
Hematocrit (HCT)		Coef (95CI)		Coef (95CI)
	Shelter: Intra-domicile		Sterilized: No	ref
	Peri-domicile	−1.77 (−3.93–0.38)	Yes	4.7 (2.11–7.29)
	Extra-domicile	−6.19 (−12.09 - −0.3)	Distance to open sewage	0.02 (0–0.03)
	Area: Marechal Rondon		Area: Marechal Rondon	ref
	Pau da Lima	4.65 (2.41–6.88)	Pau da Lima	−2.54 (−5.48–0.4)
	Food: Balance feed		Wall material: Concrete or covered brick	ref
	Homemade/others	−3.93 (−6.5 - −1.36)	Uncovered brick	0.08 (0.01–0.15)
	Mixed	0.56 (−2.02–3.14)		
	Age class: Adult			
	Infant	−1.23 (−5.41–2.95)		
	Juvenil	1.43 (−1.17–4.03)		
	Senile	−4.29 (−7.65 - −0.94)		
	Handling: Intradomiciliary			
	Peridomiciliary	−2.97 (−5.11 - −0.84)		
	% Impervious Landcover (50 m)	0.13 (0.04–0.21)		
	Dewormed: No			
	Yes	1.8 (−0.44–4.05)		
	Sterilized: No			
	Yes	2.38 (−1.26–6.03)		
total protein (PPT)	Food: Balance feed	ref	Sterilized: No	
	Homemade/others	−0.04 (−0.46–0.37)	Yes	0.35 (−0.05–0.76)
	Mixed	0.47 (0.08–0.86)	Handling: Intradomiciliary	
	Daily garbage collection: No	ref	Peridomiciliary	0.34 (−0.05–0.73)
	Yes	−0.58 (−0.98 - −0.17)	Residents per room	−0.18 (−0.36 - −0.01)
	Age class: Adult	ref	Food: Balance feed	
	Infant	−1.02 (−1.68 - −0.37)	Homemade/others	−0.7 (−1.41–0.01)
	Juvenil	−0.62 (−1.03 - −0.21)	Mixed	0.06 (−0.38–0.49)
	Senile	1.19 (0.67–1.72)	% Impervious Landcover (50 m)	0.01 (0–0.02)
	Handling: Intradomiciliary	ref	CCZ activity: <1 yr	
	Peridomiciliary	0.34 (0–0.67)	> 1 yr	−0.33 (−0.74–0.07)
			never	0.01 (−0.43–0.45)
leukocyte count (WBC)	Sterilized: No	ref	Shelter: Intra-domicile	
	Yes	−2287.42 (−4387.87 - −186.97)	Peri-domicile	901.90 (−2582.44–4386.2588)
	Shelter: Intra-domicile	ref	Extra-domicile	7581.21 (1662.31–13500.10)
	Peri-domicile	966.13 (−297.54–2229.79)	Pavimented access: No	
	Extra-domicile	−174.35 (−3178.88–2830.19)	Yes	3610.35 (−213.84–7434.55)
			Sterilized: No	
			Yes	−3571.84 (−7200.28–56.59)
			Sex: Female	
			Male	−2712.09 (−5880.40–456.21)
			Handling: Intradomiciliary	
			Peridomiciliary	2492.88 (−940.05–5925.82)
Neutrophils (Neut)	Sterilized: No	ref	Sterilized: No	
	Yes	−1227.15 (−2659.76–205.46)	Yes	−3275.15 (−6070.2 - −480.1)
	Yard material: Covered	ref	Shelter: Intra-domicile	
	Not Yard	855.43 (−94.83–1805.68)	Peri-domicile	590.62 (−2038.9–3220.13)
	Uncovered	256.1 (−729.94–1242.15)	Extra-domicile	7995.36 (3164.03–12826.69)
Lymphocytes (Lym)	Area: Marechal Rondon	ref	Daily garbage collection: No	ref
	Pau da Lima	852.45 (129.71–1575.19)	Yes	−1352.69 (−2836.82–131.44)
	Daily garbage collection: No	ref	Sex: Female	ref
	Yes	−641.08 (−1485.04–202.88)	Male	−1634.14 (−2863.07 - −405.21)
			Peridomestic area: No	ref
			Yes	1125.71 (−339.34–2590.76)
			Age class: Adult	ref
			Infant	1323.18 (−740.19–3386.55)
			Juvenil	1061.32 (−429.68–2552.32)
			Senile	−1543.56 (−3979.42–892.29)
Eosinophils (Eos)	Age class: Adult		Dewormed: No	ref
	Infant	756.42 (74.93–1437.91)	Yes	−435.04 (−902.06–31.98)
	Juvenil	−259.6 (−701.27–182.06)		
	Senile	−398.89 (−938.17–140.39)		
	Wall material: Concrete or covered brick			
	Uncovered brick/other	454.09 (−146.77–1054.95)		
	% Impervious Landcover (20 m)	−10.62 (−21.17 - −0.07)		
	Sex: Female			
	Male	−363.25 (−702.74 - −23.77)		
	Food: Balance feed			
	Homemade/others	65.74 (−365.96–497.43)		
	Mixed	−387.92 (−840.6–64.76)		
	Residents per room	107.11 (−84.35–298.56)		
	Daily garbage collection: No			
	Yes	172.66 (−240.83–586.14)		
Ancylostoma	Handling: Intradomiciliary		Shelter: Intra-domicile	ref
	Peridomiciliary	2.94 (1.23–7.07)	Peri-domicile	14.15 (1.24–162.12)
	% Impervious Landcover (50 m)	0.97 (0.94–1)	Handling: Intradomiciliary	ref
	Dewormed: No		Peridomiciliary	5 (0.39–63.45)
	Yes	0.47 (0.2–1.14)		
	Residents per room	1.37 (0.87–2.17)		

The variables that contributed to an increase in total plasma protein (PPT) in dogs included ingestion of a mix of homemade and commercial food [coef = 0.47 (95% CI: 0.08–0.86)], animals under semi-domiciliary management [coef = 0.34 (95% CI: 0.00–0.67)], and those in the senile age group [coef = 1.19 (95% CI: 0.67–1.72)]. Conversely, daily garbage collection [coef = −0.58 (95% CI: −0.98 - −0.17)], and the puppy [coef = −1.02 (95% CI: −1.68 - −0.37)] and juvenile [coef = −0.62 (95% CI: −1.03 - −0.21)] age groups contributed to a reduction in PPT. For cats, the category of homemade food [coef = −0.70 (95% CI: −1.41–0.01)] and the number of residents per room [coef = −0.18 (95% CI: −0.36 - −0.01)] were significantly associated with a decrease in total plasma protein values.

Neutering was negatively associated with changes in the white blood cell (WBC) count of dogs [coef = −2287.42 (95% CI: −4387.87 to −186.97)], and location of shelter was also relevant to the model. Conversely, cats sleeping outside the home were associated with an increase in WBC count [coef = 7581.21 (95% CI: 1662.31 to 13500.10)]. To be sterilized and type of yard were the relevant variables in neutrophil count in dogs, although without statistical significance. For cats, being neutered [coef = −3275.15 (95% CI: −6070.20 to −480.10)] and sheltering outside the home [coef = 7995.36 (95% CI: 3164.03 to 12826.69)] were categories of relevance for this count.

In dogs, living in Pau da Lima [coef = 852.45 (95% CI: 129.71 to 1575.19)] and in cats, being male [coef = −1634.14 (95% CI: −2863.07 to −405.21)] were relevant factors associated with variations in lymphocyte count. For dogs, being a puppy [coef = 756.42 (95% CI: 74.93 to 1437.91)] was significantly associated with an increase in eosinophil count, while male sex [coef = −363.25 (95% CI: −702.74 to −23.77)] and the percentage of impermeable ground cover within a 20 m radius [coef = −10.62 (95% CI: −21.17 to −0.07)] were associated with a reduction in this count. History of deworming was a factor negatively associated with eosinophil count only in cats [coef = −435.04 (95% CI: −902.06 to −31.98)].

Dog management was a factor associated with hookworm positivity in semi-domiciled dogs [coef = 2.94 (95% CI: 1.23–7.07)]. In cats, shelter in the peridomicile [coef = 14.15 (95% CI: 1.24–162.12)] indicated an increased chance of positivity.

**Fig. 4:**
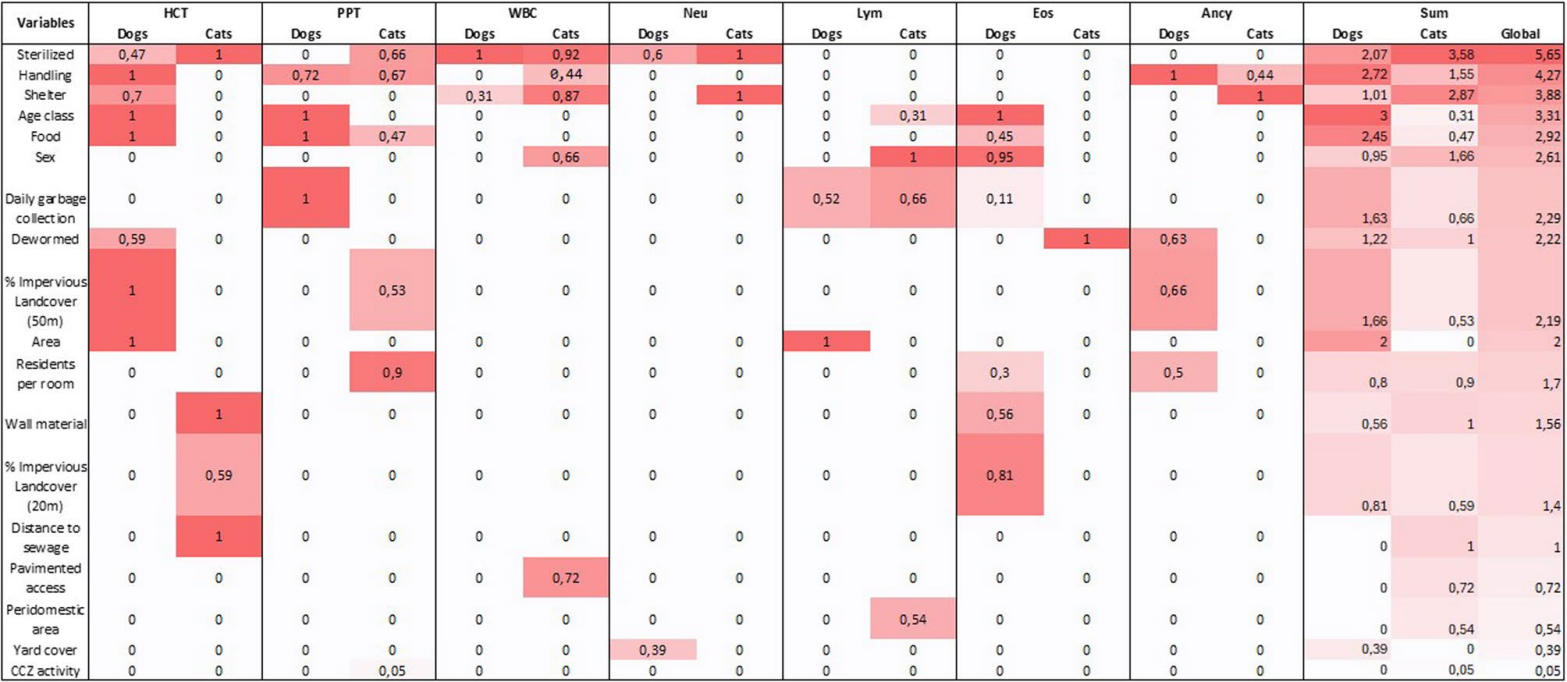
Heatmap of the relative relevance of the variables obtained as a result of the multimodel approach for each of the health parameters analyzed. The variables are ranked based on the sum of the relative relevance values.Legend: hematocrit (HCT), total protein (PPT), leukocyte count (WBC), neutrophil count (Neu), lymphocyte count (Lym), eosinophil count (Eos), positive for Ancylostoma (Ancy) and sum of Akaike weights (Sum). The intensity of the reds is associated with the relative importance of the variable for each of the models and for the sum of the models overall and by species

When we examined the sum of the weights of the variables from the different averaged models relevant to these associations, we found that the five most relevant variables overall were sterilization, type of management, shelter, age, and diet (Fig. 4; see Additional file 2).

## Discussion

In our study, we have demonstrated that dogs and cats, living with their guardians in impoverished urban informal settlements in Salvador, Brazil, have haematological signs of poorer health and greater evidence of parasitological and infectious disease burdens than a reference group admitted to a private veterinary hospital in Salvador during the same period for elective non-infectious procedures or vaccination. We further demonstrated how individual animal variables, household characteristics, and the environment in these communities are associated with these changes in haematological and parasitological tests and hence may account for them.

The changes in red series and total plasma protein in the animals studied can be interpreted as a result of nutritional, inflammatory, and infectious processes, dehydration, chronic metabolic alterations, among others. In contexts of socio-economic vulnerability, obtaining a balanced diet is a challenge for both humans and animals, directly impacting red blood cell production [26, 27]. It is well described that parasitic and infectious agents in dogs and cats can increase plasma protein levels through immune response [28, 29]. In the context of community animals, exposure to infectious agents from the environment (proximity to garbage, synanthropic reservoirs, and increased presence of vectors) and through management practices (unrestricted access to the street) potentiates contact with pathogens, associated with changes in TPP. In addition, the consumption of nutritionally poor and pro-inflammatory foods exacerbates these effects [30]. Undiagnosed chronic diseases, such as kidney and liver failure [31] and hemoparasitosis [32], can also contribute to increased total proteins. Furthermore, aside from inflammatory and infectious processes, this increase in TPP can indicate dehydration [33]. Water deprivation can occur during periods when animals are wandering (semi-domesticated) or even tethered in peridomestic areas, where exposure to heat subjects them to thermal stress and increases water loss.

We observed that WBC was found to be correlated with neutering and shelter location in dogs and cats, and in cats also with sex, type of management and whether the entrance to the home was paved. According to Boone [33], physiological, pathological, or pharmacological changes can result in alterations in white blood cells. Therefore, an increase in the number of circulating leukocytes (leukocytosis) typically indicates an acute immune response, whereas a decrease (leukopenia) suggests immune suppression. In vulnerable animals, the presence of parasites can be a cause of leukocytosis due to an increase in eosinophils. Geohelminths, mites, fleas, and ticks can trigger eosinophilic responses in these animals [34]. Apart from parasitic conditions, food allergies associated with the type of food offered, or inflammation [35] could also contribute to leukocytosis. We hypothesize that the leukocytosis observed in community cats may be associated with stress and inflammation, potentially leading to increases in neutrophils and lymphocytes. These responses are indicative of the immune system’s reaction to various environmental and physiological stressors encountered in their living conditions [36].

The presence of parasitized and infested animals within communities is a prevalent issue, contributing significantly to the maintenance of helminth lifecycles (e.g. *Ancylostoma* spp.) and the transmission of ectoparasites such as ticks, fleas, and mites. Stray animals, which are devoid of any form of care, frequently interact with semi-domesticated animals, thereby facilitating a continuous cycle of infection and infestation [37]. The financial burden associated with deworming and the lack of adherence to animal welfare practices further perpetuate these parasitic cycles. Enforcing individual responsibility is particularly challenging, as many pet guardians lack the necessary resources to provide adequate care for their animals. This socioeconomic barrier exacerbates the issue, impeding the implementation of effective control measures, limiting the immune response capacity of individuals, perpetuating a vicious cycle of infection maintenance and increasing the load of parasitic and infectious pathogens in the system [38].

Environmental variables such as soil cover type, proximity to sewers, frequency of garbage collection, and wall material appeared to significantly influence some of the investigated parameters. Without advancements in sanitation and infrastructure in these vulnerable urban communities, controlling reservoir animals and maintaining their quality of life will remain challenging.

Several studies have highlighted the influence of individual factors such as breed, sex, age, and nutrition, as well as environmental factors like altitude, on hematological parameters in animals [39, 40]. Notably, we were able to identify socio-environmental and management variables associated with these health parameters alterations, besides the classic individual features. Interestingly, among the most relevant variables, such as being sterilised, type of management, shelter location and type of feeding could be targets of intervention strategies in order to improve the health and reduce the competences as hosts of infectious disease of domestic animals in marginalized communities. Animal welfare, the human-animal relationship, and its consequences should be the focus of a health education process, placing the responsibility on public institutions to promote, implement, and strengthen animal welfare programs and policies. Additionally, studies on reservoirs in urban centers should increasingly discuss the socioeconomic and environmental factors that underlie the process, thereby shifting the focus away from the pathogens themselves. We believe that progress in animal welfare and public health policies would positively impact the health outcomes of both animals and humans living in vulnerable communities. Therefore, an interconnected approach is necessary, allowing solutions to encompass both human and animal factors.

Our study has several limitations that should be acknowledged. First, clinical information was based primarily on owner questionnaires and physical examinations. Chronic conditions, previous diseases, or trauma were not systematically investigated due to financial constraints, and we recognize their potential impact on hematological interpretation. However, if untreated underlying diseases were present, this would further highlight the limited access of this population to veterinary care. Body condition scoring was conducted according to the guidelines of the World Small Animal Veterinary Association (WSAVA) [41]. The breed was not considered as a cofactor, although approximately 82.6% of the participating animals were mixed-breed, a category highly prevalent among companion animals in Brazil. We therefore consider it unlikely that breed exerted a significant influence on our hematological data. Physiological factors such as age, sex, and reproductive status were partially accounted for in the models but could not be fully controlled. Finally, the cross-sectional design restricts causal inference and allows only the identification of associations between socioenvironmental variables and health outcomes. Nevertheless, the study provides robust evidence of the relationship between indicators of vulnerability, including nutrition, management, and sanitation, and animal health, reinforcing the importance of targeted One Health interventions. Our sampling strategy was not random, given we aimed to do a census in the delineated study area, although not all households with animals were recruited. Disinterest in participating in the study, observed in some guardians, was directly associated with a lack of perceived importance for animal health, inability to manage their animals due to aggression, absence of immediate compensation, or insufficient time to complete the questionnaire and accompany the team during sample collection, as all procedures were required to be conducted under the supervision of the guardian. Voluntary participation of community members may have introduced selection bias, as individuals who were more engaged or had a greater awareness of the importance of the topic tended to collaborate more actively. In addition, lack of interest, limited time, or unavailability of some participants may have reduced the representativeness of the sample in relation to the overall target population.

## Conclusion

In summary, the laboratory abnormalities observed in animals from the community could potentially be mitigated through basic animal health interventions, such as sterilization, improved nutrition, deworming, and limiting their access to the streets. While implementing these measures may reduce the competence of dogs and cats as host for infectious agents, including zoonotic pathogens, our study did not directly assess pathogen transmission or zoonotic risk. The vulnerable conditions faced by these communities may hinder access to such essential health interventions, highlighting the importance of developing public policies aimed at promoting and protecting animal health. Beyond improving the well-being of companion animals, these initiatives could also have important implications for public health by potentially reducing zoonotic diseases risk and supporting overall community health.

## Supplementary Information

Supplementary material 1. Additional file 1: Multimodel results description. Model sets and loglikelihood, AIC, deltaAIC and weight for each model combination with a delta AIC <2. Also the sum of weights of each variable included in the averaged models.

Supplementary material 2.

## Data Availability

No datasets were generated or analysed during the current study.
