# Supplementary material for: Socioeconomic vulnerability and the management of domestic animal hosts in urban environments: a one health issue
Source: BMC Vet Res. 2025 Dec 13;21:699. doi: 10.1186/s12917-025-05062-7 (PMC12702158; doi:10.1186/s12917-025-05062-7)
Supplement: Supplementary file 1 — Supplementary material 1. Additional file 1: Multimodel results description. Model sets and loglikelihood, AIC, deltaAIC and weight for each model combination with a delta AIC <2. Also the sum of weights of each variable included in the averaged models. [file 12917_2025_5062_MOESM1_ESM.docx]

Household Survey - Animal Census

### Variable / Field Name

**Field Label**

**Field Attributes (Field Type, Validation, Choices, Calculations, etc.)**

- **[cd_us1]** Interviewer Username - AUTOMATIC RESPONSE

- Text, Required

- Annotation: @USERNAME or @APPUSERNAME, HIDDEN

- **[ent_dat]** Interview Date:

- Text (date_dmy), Required

- **[bairro_ad]** Neighborhood of the house (Animals):

- Radio, Required

- Example: Marechal Rondon

- **[quartnum_ad]** House number on the block (Animals):

- Text (number, Min: 10, Max: 999), Required

- **[usod_ad]** Usage of the household:

- Radio, Required

- 1 Empty

- 2 Abandoned

- 3 Under construction

- 4 Commercial

- 5 Residential

- 6 Both Commercial and Residential

- **[quart_ad]** House block (01 - 99) (Animals):

- Text (integer, Min: 1, Max: 99), Required

- **[seg_ad]** Follow-up:

- Radio, Required

- 1 Accepted

- 2 Refused

- 3 Not found

- 4 Access blocked

- **[mora4]** Number of residents in the house?

- Text (number, Min: 1, Max: 25), Required

- **[quart14]** Number of rooms?

- Text (number, Min: 0, Max: 7), Required

- **[area14]** Does the house have a peri-domestic area?

- Radio, Required

- 1 Yes, external area

- 0 No, no external area

- **[area_s1]** If yes, what type?

- Radio, Required

- 1 Backyard

- 2 Terrace

- 3 Covered area

- **[casa2]** Is the access to your house paved?

- Radio, Required

- 1 Yes, paved

- 0 No, dirt/grass

- **[alaga1]** How does the resident prevent flood water from entering the house?

- Radio, Required

- 1 Closed wall

- 2 Threshold at the entrance to the yard

- 3 Threshold at the entrance to the house

- 4 Raised house floor

- 5 Lives on a higher floor

- 0 None

- **[material1]** What is the predominant material of the house walls?

- Radio, Required

- 1 Concrete or covered brick

- 2 Uncovered brick

- 3 Wood or other non-masonry material

- **[quintal3]** Is the backyard of your house covered?

- Radio, Required

- 1 Yes, covered

- 0 No, dirt/grass

- 9 Not applicable (no backyard)

- **[imovel1]** Is the property located on a slope?

- Radio, Required

- 1 Yes

- 0 No

- **[descarta47]** How do you dispose of your trash?

- Radio, Required

- 1 Only plastic bags

- 2 Only containers with lids

- 3 Both

- 4 Others

- **[outros14]** If others, specify:

- Text, Required

- **[coleta1]** Where do you place your trash for collection?

- Radio, Required

- 1 Directly in front of the house

- 2 Elevated

- 3 Collective container

- 4 Vacant lot

- 5 Others

- **[freque12]** Frequency of trash collection:

- Radio, Required

- 1 Daily

- 2 Once a week

- 3 Twice a week

- 4 Three times a week

- **[animais1]** Animals at home?

- Radio

- 1 Yes

- 0 No

- **[especie1]** Species:

- Checkbox, Required

- 1 Cat

- 2 Dog

- 3 Chicken

- 4 Others

- **[gato_12]** Number of cats:

- Text (number), Required

- **[caes1]** Number of dogs:

- Text (number), Required

- **[frang]** Number of chickens:

- Text (number), Required

- **[anims]** If other animals, specify:

- Text, Required

- **[ccz1]** How long since the last rodent control activity by the CCZ at your home/street?

- Radio, Required

- 1 Months

- 2 More than a year

- 3 Never

- **[agent]** Number of health agent visits in the last six months?

- Text (number), Required

- **[casanova_ad]** House identification number (Domestic Animal):

- Text (number, Min: 501010, Max: 599999), Required

- **[erro1]** ERROR: CASANOVA does not match the entered information!

- Please verify the block and house number.

---

**Animal Data Collection and Health Evaluation**

- **[id_animal]** Animal Identification (ONLY Domestic Animals):

- Text

- **[apelido]** Animal Nickname:

- Text, Required

- **[animal_dom]** Animal Species (ONLY Domestic Animals):

- Radio, Required

- 1 Dog

- 2 Cat

- 3 Cattle

- 4 Horse

- 5 Pig

- 6 Bird

- 7 Others

- **[outros_id_anim]** If others, specify:

- Text, Required

---

**Consent and Sample Collection**

- **[consent_ad]** Owner consented to the animal’s participation in the study?

- Radio, Required

- 1 Yes

- 0 No

- **[dt_consent_ad]** Consent Date (Animal):

- Text (date_dmy), Required

- **[tcle]** Was an Informed Consent Form (ICF) obtained for the animal?

- Radio, Required

- 1 Yes

- 0 No

- **[coleta_ad]** Was a blood sample collected from the animal?

- Radio, Required

- 1 Yes

- 0 No

- **[soro]** Was it collected in a serum tube?

- Radio, Required

- 1 Yes

- 0 No

- **[edta]** Was it collected in an EDTA tube?

- Radio, Required

- 1 Yes

- 0 No

- **[dtcoleta_ad]** Date of blood collection:

- Text (date_dmy), Required

- **[urina_ad]** Was a urine sample collected from the animal?

- Radio, Required

- 1 Yes

- 0 No

- **[dturina]** Date of urine collection:

- Text (date_dmy), Required

- **[fezes_ad]** Was a fecal sample collected from the animal?

- Radio, Required

- 1 Yes

- 0 No

- **[dfezes]** Date of fecal collection:

- Text (date_dmy), Required

- **[col_swaboral]** Was an oral swab collected from the animal?

- Radio, Required

- 1 Yes

- 0 No

- **[dswb_oral]** Date of oral swab collection:

- Text (date_dmy), Required

- **[col_swab_ret]** Was a rectal swab collected from the animal?

- Radio, Required

- 1 Yes

- 0 No

- **[dswb_retal]** Date of rectal swab collection:

- Text (date_dmy), Required

---

**Animal Health and Characteristics**

- **[raca_anim]** Breed:

- Text, Required

- **[sexo_anim]** Sex:

- Radio, Required

- 1 Male

- 0 Female

- 9 Unknown

- **[castrado]** Is the animal neutered?

- Radio, Required

- 1 Yes

- 0 No

- **[tem_animal]** How long has the animal been with you?

- Text (number), Required

- **[idade_anos]** Age of the animal (in years):

- Text (number)

- **[idade_anim]** Age category:

- Radio, Required

- 1 Puppy

- 2 Juvenile

- 3 Adult

- 4 Senior

---

**Health Observations and Vaccination History**

- **[alimentacao]** Feeding:

- Checkbox, Required

- 1 Dry food

- 2 Homemade food

- 3 Other

- **[abrigo]** Shelter:

- Radio, Required

- 1 Indoors

- 2 Peridomestic

- 3 Outdoors

- **[condicao_corp]** Body condition:

- Radio, Required

- 1 Normal

- 2 Thin

- 3 Cachectic

- 4 Overweight

- 5 Obese

- **[vacinado]** Vaccinated:

- Radio, Required

- 1 Yes

- 0 No

- **[cani_vac]** Dog Vaccines:

- Checkbox, Required

- 1 Canine Multivalent

- 2 Rabies

- 3 Other

- **[felino]** Cat Vaccines:

- Radio, Required

- 1 Cat Multivalent

- 2 Rabies

- 3 Other

---

**Parasite Control and Physical Exam**

- **[vermifugacao]** Dewormed:

- Radio, Required

- 1 Yes

- 0 No

- **[cont_ani]** Does the animal have contact with non-domestic animals (e.g., bats, pigeons, rats)?

- Radio, Required

- 1 Yes

- 0 No

- **[cobras]** Has the animal had any incidents with scorpions or snakes?

- Radio, Required

- 1 Yes

- 0 No

- **[peri_domic]** Animals seen in the peri-domestic area:

- Checkbox, Required

- 1 Marmoset

- 2 Bat

- 3 Opossum

- 4 Rat

- 5 Others
