# Supplementary material for: Socioeconomic vulnerability and the management of domestic animal hosts in urban environments: a one health issue
Source: BMC Vet Res. 2025 Dec 13;21:699. doi: 10.1186/s12917-025-05062-7 (PMC12702158; doi:10.1186/s12917-025-05062-7)
Supplement: Supplementary file 2 — Supplementary material 2. [file 12917_2025_5062_MOESM2_ESM.docx]

Additional Files

**Additional Files 2**: Multimodel results description. Model sets and loglikelihood, AIC, deltaAIC and weight for each model combination with a delta AIC <2. Also the sum of weights of each variable included in the averaged models.

| Canines |  | |  |  |  |  |  |  |
| --- | --- | --- | --- | --- | --- | --- | --- | --- |
| PPT | Component models: | |  |  |  |  |  |  |
|  |  | df | logLik | AICc | delta | weight |  |  |
|  | 1234 | 9 | -296.37 | 611.73 | 0 | 0.72 |  |  |
|  | 123 | 8 | -298.43 | 613.64 | 1.91 | 0.28 |  |  |
|  |  |  |  |  |  |  |  |  |
|  |  |  |  |  |  |  |  |  |
|  |  |  |  |  |  |  |  |  |
|  | Term codes: | Food | Garb_freq | Age class | manejo |  |  |  |
|  |  | 1 | 2 | 3 | 4 |  |  |  |
|  | Sum of weights: | 1 | 1 | 1 | 0.72 |  |  |  |
|  | N containing models: | 2 | 2 | 2 | 1 |  |  |  |
|  |  |  |  |  |  |  |  |  |
| Hematocrit | Component models: | |  |  |  |  |  |  |
|  |  | df | logLik | AICc | delta | weight |  |  |
|  | 1245678 | 13 | -784.91 | 1597.51 | 0 | 0.24 |  |  |
|  | 124567 | 12 | -786.29 | 1598.01 | 0.5 | 0.18 |  |  |
|  | 12345678 | 14 | -784.29 | 1598.54 | 1.02 | 0.14 |  |  |
|  | 1234567 | 13 | -785.45 | 1598.58 | 1.07 | 0.14 |  |  |
|  | 245678 | 11 | -787.94 | 1599.08 | 1.57 | 0.11 |  |  |
|  | 2345678 | 12 | -786.87 | 1599.17 | 1.66 | 0.1 |  |  |
|  | 234567 | 11 | -788.1 | 1599.41 | 1.89 | 0.09 |  |  |
|  |  |  |  |  |  |  |  |  |
|  | Term codes: | Shelter | Area | Sterilized | Food | Age class | manejo | % Impervious Landcover |
|  |  | 1 | 2 | 3 | 4 | 5 | 6 | 7 |
|  | Sum of weights: | 0.7 | 1 | 0.47 | 1 | 1 | 1 | 1 |
|  | N containing models: | 4 | 7 | 4 | 7 | 7 | 7 | 7 |
|  |  |  |  |  |  |  |  |  |
| WBC | Component models: | |  |  |  |  |  |  |
|  |  | df | logLik | AICc | delta | weight |  |  |
|  | 2 | 3 | -2232.09 | 4470.3 | 0 | 0.69 |  |  |
|  | 12 | 5 | -2230.79 | 4471.86 | 1.56 | 0.31 |  |  |
|  |  |  |  |  |  |  |  |  |
|  | Term codes: | Shelter | Sterilized |  |  |  |  |  |
|  |  | 1 | 2 |  |  |  |  |  |
|  | Sum of weights: | 0.31 | 1 |  |  |  |  |  |
|  | N containing models: | 1 | 2 |  |  |  |  |  |
|  |  |  |  |  |  |  |  |  |
|  |  |  |  |  |  |  |  |  |
|  |  |  |  |  |  |  |  |  |
| Neutrophils | Component models: | |  |  |  |  |  |  |
|  |  | df | logLik | AICc | delta | weight |  |  |
|  | 1 | 3 | -2002.82 | 4011.75 | 0 | 0.37 |  |  |
|  | (Null) | 2 | -2004.28 | 4012.61 | 0.86 | 0.24 |  |  |
|  | 12 | 5 | -2001.19 | 4012.67 | 0.92 | 0.23 |  |  |
|  | 2 | 4 | -2002.59 | 4013.36 | 1.62 | 0.16 |  |  |
|  |  |  |  |  |  |  |  |  |
|  | Term codes: | Sterilized | Yard cover |  |  |  |  |  |
|  |  | 1 | 2 |  |  |  |  |  |
|  | Sum of weights: | 0.6 | 0.39 |  |  |  |  |  |
|  | N containing models: | 2 | 2 |  |  |  |  |  |
|  |  |  |  |  |  |  |  |  |
| Limphocytes | Component models: | |  |  |  |  |  |  |
|  | 12 | 4 | -2045.87 | 4099.93 | 0 | 0.52 |  |  |
|  | 1 | 3 | -2047 | 4100.12 | 0.19 | 0.48 |  |  |
|  |  |  |  |  |  |  |  |  |
|  | Term codes: | Area | Daily garbage collection |  |  |  |  |  |
|  |  | 1 | 2 |  |  |  |  |  |
|  | Sum of weights: | 1 | 0.52 |  |  |  |  |  |
|  | N containing models: | 2 | 1 |  |  |  |  |  |
|  |  |  |  |  |  |  |  |  |
| Eosinophils | Component models: | |  |  |  |  |  |  |
|  |  | df | logLik | AICc | delta | weight |  |  |
|  | 357 | 8 | -1786.37 | 3589.46 | 0 | 0.16 |  |  |
|  | 37 | 7 | -1787.62 | 3589.8 | 0.34 | 0.13 |  |  |
|  | 567 | 5 | -1790.17 | 3590.63 | 1.17 | 0.09 |  |  |
|  | 3567 | 9 | -1786 | 3590.91 | 1.45 | 0.08 |  |  |
|  | 3457 | 10 | -1784.9 | 3590.91 | 1.46 | 0.08 |  |  |
|  | 237 | 10 | -1784.92 | 3590.95 | 1.49 | 0.08 |  |  |
|  | 3 | 6 | -1789.3 | 3591.01 | 1.55 | 0.07 |  |  |
|  | 367 | 8 | -1787.23 | 3591.18 | 1.72 | 0.07 |  |  |
|  | 35 | 7 | -1788.34 | 3591.23 | 1.77 | 0.07 |  |  |
|  | 4567 | 7 | -1788.39 | 3591.34 | 1.88 | 0.06 |  |  |
|  | 1357 | 9 | -1786.25 | 3591.4 | 1.94 | 0.06 |  |  |
|  | 137 | 8 | -1787.35 | 3591.42 | 1.97 | 0.06 |  |  |
|  |  |  |  |  |  |  |  |  |
|  | Term codes: | Food | Daily garbage collection | Age class | Wall material | Residents per room | % Impervious Landcover | Sex |
|  |  | 1 | 2 | 3 | 4 | 5 | 6 | 7 |
|  | Sum of weights: | 0.45 | 0.11 | 1 | 0.56 | 0.3 | 0.81 | 0.95 |
|  | N containing models: | 7 | 2 | 14 | 8 | 5 | 11 | 13 |
|  |  |  |  |  |  |  |  |  |
| Ancylostoma | Component models: | |  |  |  |  |  |  |
|  |  | df | logLik | AICc | delta | weight |  |  |
|  | 134 | 4 | -63.44 | 135.21 | 0 | 0.21 |  |  |
|  | 13 | 3 | -64.76 | 135.72 | 0.51 | 0.16 |  |  |
|  | 1234 | 5 | -62.63 | 135.78 | 0.57 | 0.16 |  |  |
|  | 124 | 4 | -63.89 | 136.11 | 0.91 | 0.13 |  |  |
|  | 14 | 3 | -65.02 | 136.23 | 1.03 | 0.13 |  |  |
|  | 123 | 4 | -63.96 | 136.25 | 1.04 | 0.13 |  |  |
|  | 12 | 3 | -65.47 | 137.15 | 1.94 | 0.08 |  |  |
|  |  |  |  |  |  |  |  |  |
|  | Term codes: | Handling | Residents per room | % Impervious Landcover (50m) | Dewormed |  |  |  |
|  |  | 1 | 2 | 3 | 4 |  |  |  |
|  | Sum of weights: | 1 | 0.5 | 0.66 | 0.63 |  |  |  |
|  | N containing models: | 7 | 4 | 4 | 4 |  |  |  |
| Felines |  |  |  |  |  |  |  |  |
|  |  | df | logLik | AICc | delta | weight |  |  |
| PPT | 145 | 5 | -58.49 | 128.1 | 0 | 0.1 |  |  |
|  | 1345 | 7 | -56.02 | 128.2 | 0.1 | 0.1 |  |  |
|  | 156 | 5 | -58.74 | 128.58 | 0.49 | 0.08 |  |  |
|  | 1456 | 6 | -57.55 | 128.68 | 0.59 | 0.08 |  |  |
|  | 356 | 6 | -57.59 | 128.77 | 0.67 | 0.08 |  |  |
|  | 345 | 6 | -57.61 | 128.81 | 0.71 | 0.07 |  |  |
|  | 56 | 4 | -60.05 | 128.83 | 0.73 | 0.07 |  |  |
|  | 3456 | 7 | -56.49 | 129.13 | 1.03 | 0.06 |  |  |
|  | 134 | 6 | -57.85 | 129.28 | 1.18 | 0.06 |  |  |
|  | 456 | 5 | -59.17 | 129.44 | 1.34 | 0.05 |  |  |
|  | 13456 | 8 | -55.33 | 129.48 | 1.38 | 0.05 |  |  |
|  | 1356 | 7 | -56.67 | 129.5 | 1.41 | 0.05 |  |  |
|  | 1245 | 7 | -56.71 | 129.58 | 1.48 | 0.05 |  |  |
|  | 15 | 4 | -60.55 | 129.83 | 1.73 | 0.04 |  |  |
|  | 14 | 4 | -60.64 | 130.01 | 1.91 | 0.04 |  |  |
|  |  |  |  |  |  |  |  |  |
|  | Term codes: | Sterilized | CCZ activity | Food | Handling | Residents per room | % Impervious Landcover (50m) |  |
|  |  | 1 | 2 | 3 | 4 | 5 | 6 |  |
|  | Sum of weights: | 0.66 | 0.05 | 0.47 | 0.67 | 0.9 | 0.53 |  |
|  | N containing models: | 10 | 1 | 7 | 10 | 13 | 8 |  |
|  |  |  |  |  |  |  |  |  |
|  |  | df | logLik | AICc | delta | weight |  |  |
| Hematocrit | 1234 | 6 | -210.38 | 434.05 | 0 | 0.59 |  |  |
|  | 124 | 5 | -211.94 | 434.8 | 0.75 | 0.41 |  |  |
|  |  |  |  |  |  |  |  |  |
|  | Term codes: | Sterilized | Distance to sewage | Wall material | % Impervious Landcover |  |  |  |
|  |  | 1 | 2 | 3 | 4 |  |  |  |
|  | Sum of weights: | 1 | 1 | 1 | 0.59 |  |  |  |
|  | N containing models: | 2 | 2 | 2 | 1 |  |  |  |
|  |  |  |  |  |  |  |  |  |
|  |  | df | logLik | AICc | delta | weight |  |  |
| WBC | 1235 | 7 | -751.66 | 1519.03 | 0 | 0.17 |  |  |
|  | 12345 | 8 | -750.66 | 1519.53 | 0.51 | 0.13 |  |  |
|  | 123 | 6 | -753.33 | 1519.92 | 0.89 | 0.11 |  |  |
|  | 1234 | 7 | -752.14 | 1519.97 | 0.94 | 0.11 |  |  |
|  | 125 | 6 | -753.67 | 1520.59 | 1.56 | 0.08 |  |  |
|  | 135 | 6 | -753.67 | 1520.6 | 1.57 | 0.08 |  |  |
|  | 134 | 6 | -753.78 | 1520.82 | 1.79 | 0.07 |  |  |
|  | 1345 | 7 | -752.57 | 1520.84 | 1.82 | 0.07 |  |  |
|  | 235 | 5 | -754.99 | 1520.85 | 1.83 | 0.07 |  |  |
|  | 2345 | 6 | -753.81 | 1520.88 | 1.85 | 0.07 |  |  |
|  | 13 | 5 | -755.05 | 1520.99 | 1.97 | 0.06 |  |  |
|  |  |  |  |  |  |  |  |  |
|  | Term codes: | Shelter | Pavimented access | Sterilized | Handling | Sex |  |  |
|  |  | 1 | 2 | 3 | 4 | 5 |  |  |
|  | Sum of weights: | 0.87 | 0.72 | 0.92 | 0.44 | 0.66 |  |  |
|  | N containing models: | 9 | 7 | 10 | 5 | 7 |  |  |
|  |  |  |  |  |  |  |  |  |
| Neutrophils |  | df | logLik | AICc | delta | weight |  |  |
|  | 12 | 5 | -769.87 | 1550.59 | 0 | 1 |  |  |
|  |  |  |  |  |  |  |  |  |
|  | Term codes: | Sterilized | Shelter |  |  |  |  |  |
|  |  | 1 | 1 |  |  |  |  |  |
|  | Sum of weights: | 1 | 1 |  |  |  |  |  |
|  | N containing models: | 1 | 1 |  |  |  |  |  |
|  |  |  |  |  |  |  |  |  |
|  | Component models: | |  |  |  |  |  |  |
| Linphocytes |  | df | logLik | AICc | delta | weight |  |  |
|  | 24 | 4 | -632.01 | 1272.65 | 0 | 0.26 |  |  |
|  | 124 | 5 | -631.19 | 1273.34 | 0.69 | 0.18 |  |  |
|  | 14 | 4 | -632.61 | 1273.84 | 1.19 | 0.14 |  |  |
|  | 1234 | 8 | -627.84 | 1274.08 | 1.43 | 0.12 |  |  |
|  | 4 | 3 | -633.99 | 1274.34 | 1.69 | 0.11 |  |  |
|  | 234 | 7 | -629.4 | 1274.63 | 1.98 | 0.09 |  |  |
|  | 134 | 7 | -629.4 | 1274.63 | 1.98 | 0.09 |  |  |
|  |  |  |  |  |  |  |  |  |
|  | Term codes: | Peridomestic area | Daily garbage collection | Age class | Sex |  |  |  |
|  |  | 1 | 2 | 3 | 4 |  |  |  |
|  | Sum of weights: | 0.54 | 0.66 | 0.31 | 1 |  |  |  |
|  | N containing models: | 4 | 4 | 3 | 7 |  |  |  |
|  |  |  |  |  |  |  |  |  |
| Ancylostoma |  | df | logLik | AICc | delta | weight |  |  |
|  | 1 | 2 | -15.47 | 35.39 | 0 | 0.56 |  |  |
|  | 12 | 3 | -14.46 | 35.85 | 0.46 | 0.44 |  |  |
|  |  |  |  |  |  |  |  |  |
|  | Term codes: | Shelter | Handling |  |  |  |  |  |
|  |  | 1 | 2 |  |  |  |  |  |
|  | Sum of weights: | 1 | 0.44 |  |  |  |  |  |
|  | N containing models: | 2 | 1 |  |  |  |  |  |
